# Supervised and unsupervised learning reveal heroin-induced impairments in astrocyte structural plasticity

**DOI:** 10.1126/sciadv.ads6841

**Published:** 2025-04-30

**Authors:** Michela Marini, Yabo Niu, Heng Zhao, Anish Mohan, Nathan Koorndyk, Anna Kruyer, Demetrio Labate

**Affiliations:** ^1^ Department of Mathematics, University of Houston, Houston, TX, USA.; ^2^ Division of Pharmaceutical Sciences, University of Cincinnati, Cincinnati, OH, USA.; ^3^ Neuroscience Graduate Program, University of Cincinnati, Cincinnati, OH, USA.; ^4^ Center for Addiction Research, University of Cincinnati, Cincinnati, OH, USA.

## Abstract

Astrocytes regulate synaptic activity across large brain territories via their complex, interconnected morphology. Emerging evidence supports the involvement of astrocytes in shaping relapse to opioid use through morphological rearrangements in the nucleus accumbens (NAc). However, a comprehensive assessment of astrocyte structural diversity within and between NAc subdivisions is lacking because of limitations in existing methodologies to quantify meaningful alterations in astrocyte structure. We developed a methodological pipeline that integrates supervised and unsupervised learning techniques to rigorously quantify astrocyte morphological features and spatial organization across the brain, leveraging expression of cytoskeletal markers. Application of this pipeline reveals that morphological characteristics of individual astrocytes predict their location within the NAc. Our analysis also indicates that after heroin use, astrocyte structural plasticity is impaired in portions of the NAc associated with the extinction of conditioned responses and is uniquely engaged in the dorsomedial portion of the NAc shell, an undercharacterized subdivision of the structure.

## Teaser

Machine learning reveals impaired ability of astrocytes to undergo morphological rearrangements after heroin.

## INTRODUCTION

Astrocytes are the most abundant glial type in the brain and provide metabolic and other support to enable neural circuit function (*1*, *2*). Current research highlights astrocytes as key regulators of neuronal activity, and, consequently, astrocytes are emerging as an important and highly dynamic cell type capable of shaping neural function and animal behavior (*2*, *3*). The diverse functional roles of astrocytes in the brain are enabled by their unique, highly ramified structure that permits their engagement with the vasculature, neuronal somata, synapses, and other glia simultaneously (*4*). Through these interactions, astrocytes contribute to regulation of blood flow, synaptic potentiation and depression, and recovery from injury and disease (*3*, *5*, *6*).

The strong links between changes in astrocyte structure and function in the context of neurodevelopment and disease have been supported by studies examining astrocyte cytoskeletal markers such as glial fibrillary acidic protein (GFAP) in disease models and postmortem human brain tissue, where increases or decreases in its expression in various brain nuclei are often linked with neurocognitive and psychiatric disorders. Hence, changes in GFAP expression are often the first-line test for astrocyte involvement in disease and support a role for astrocyte dysfunction in major depression, schizophrenia, alcohol and substance use disorders, anorexia nervosa, and bipolar disorder (*7*–*19*), where changes in astrocyte structure, density, complexity, and/or blood vessel association are linked with disrupted astrocyte function. Although reactive astrogliosis remains the single most studied astrocytic response involving morphological adaptations and changes in GFAP expression (*20*, *21*), in recent years, astrocyte morphological plasticity has been shown to be more nuanced. GFAP expression is dynamic across the circadian cycle (*22*–*24*) and increases with physical exercise and environmental enrichment (*25*, *26*). Moreover, in aging, astrocytes increase or decrease their GFAP expression in different brain regions (*27*, *28*), suggesting heterogeneity in astrocyte form and function.

We previously found a notable relationship between astrocyte structure and vulnerability to substance use disorders, with astrocytes in the nucleus accumbens (NAc) altering their association with different neural subcircuits to drive or suppress drug-seeking behavior depending on heroin availability (*29*–*31*). The NAc is critical for regulating behavioral outputs in response to rewards, including substances of abuse and natural reinforcers, such as food or sucrose. The NAc is composed of core and shell subregions that are themselves heterogeneous structures with regard to synaptic input and output connectivity and function (*32*–*36*). Heterogeneity has been observed in astrocyte morphology within the NAc core (*3*, *30*, *37*), but studies have not yet examined how astrocyte structure and function differ across NAc subregions at baseline or in response to operant conditioning with natural or pathological reinforcers.

To address this gap, we developed an automated pipeline for single-cell morphological analysis of astrocytes that integrates state-of-the-art deep learning models for astrocyte detection and segmentation, together with highly sensitive geometrical tools for precise quantitation of single-cell morphological characteristics. We introduce the rigorous notion of morphological distance (MD) to measure alterations in astrocyte morphology and compare astrocyte subpopulations according to their structural characteristics. By applying this pipeline in combination with supervised machine learning, we found that single-astrocyte morphological characteristics were predictive not only of anatomical location within the NAc at baseline but also of the availability of heroin or sucrose at the moment of image capture. This geometrically sensitive approach yields substantially more detailed information about astrocyte structure than previously applied manual or semiautomated approaches and serves as a rigorous quantitative assay for identifying brain nuclei where astrocytes undergo plasticity in the context of disease. We found that astrocyte structural plasticity across the NAc was disrupted in animals that had been exposed to heroin but not sucrose, consistent with a largely protective role for NAc astrocytes in maintaining synaptic homeostasis and behavioral flexibility. We also found that astrocyte structural plasticity in the dorsomedial portion of the NAc shell was uniquely engaged during the initiation of opioid but not sucrose seeking, suggesting the involvement of this structure in drug relapse.

## RESULTS

### Image processing pipeline quantified single-cell morphological characteristics

**Fig. 1.**
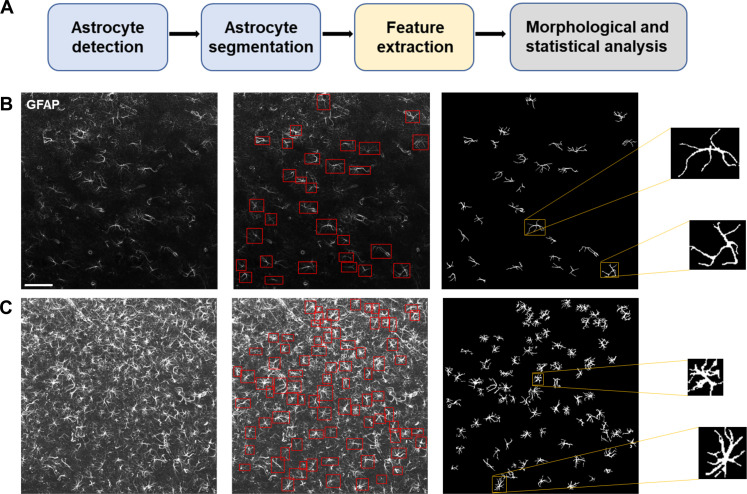
Astrocyte image analysis pipeline. The computational pipeline (**A**) processes micrographs of astrocytes by automatically detecting and segmenting astrocytes immunolabeled for GFAP before extracting image-based features useful for identifying and analyzing subpopulations defined by specific morphological properties. (**B** and **C**) From left to right, representative images from (B) the anterior core (AC) and (C) DM regions of the NAc are processed firstly to detect individual astrocytes within rectangular boxes adapted to the cell size and next to segment the same cells individually; zoom shows individual segmented cells. Scale bar, 80 μm.

**Fig. 2.**
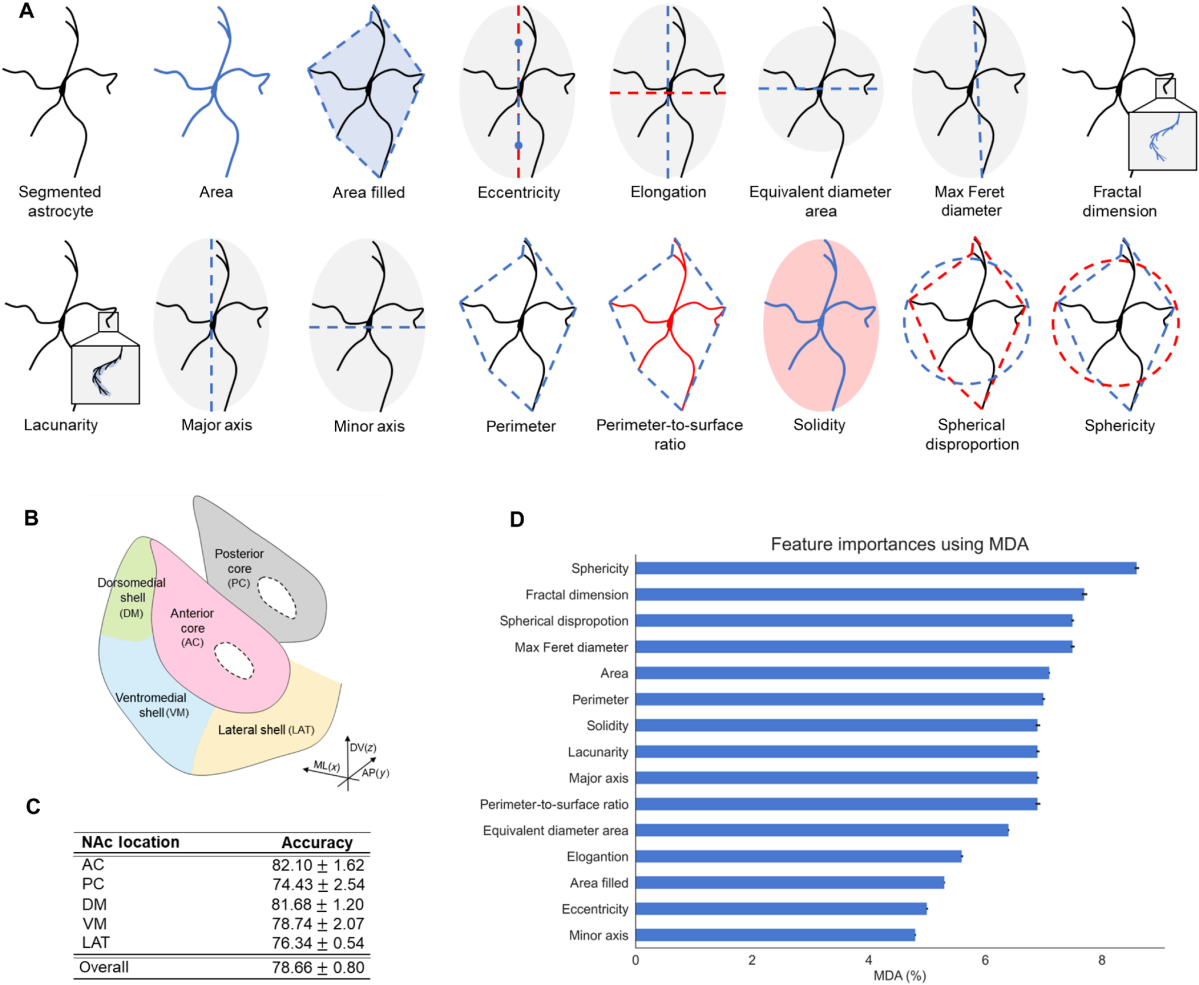
Morphological analysis of astrocytes in the NAc. (**A**) Fifteen morphological features were computed from the segmented image of an astrocyte to measure different shape characteristics. These single-cell quantities were used for training a Random Forest classifier to predict whether a blindly selected NAc astrocyte belonged to one of the five NAc subdivisions displayed in (**B**), with prediction results reported in (**C**). (**D**) The MDA measured the feature importance of the Random Forest classification model computed to predict whether a given astrocyte belonged to one of the five NAc subdivisions.

We developed an astrocyte image analysis pipeline to automatically extract and analyze single-cell phenotypic profiles of astrocytes in micrographs. The pipeline consisted of four processing units (Fig. 1A), i.e., astrocyte detection, segmentation, feature extraction and selection, and morphological and statistical modeling. The first two processing units were critical to identify cellular structures (Fig. 1B) and compute their morphometric characteristics during the subsequent single-cell level analysis. To quantify structural characteristics of astrocytes, we computed 15 single-cell morphometric features for each cell in an image, shown in Fig. 2A, selected for their interpretability and anticipated discriminatory power.

### Astrocyte structure predicted anatomical location within the NAc

We ran a numerical experiment to test the hypothesis that morphological characteristics of individual astrocytes were predictive of their location within the NAc, using anterior core (AC), posterior core (PC), dorsomedial shell (DM), ventromedial shell (VM), and lateral shell (LAT) as demarcations, as shown in Fig. 2B. We applied a multiclass Random Forest classifier and trained the classifier on the previously computed single-cell morphometric features by randomly assigning 70% of the data for training and the remaining 30% for testing. To avoid potential bias during training and test sample assignment, the data split into training and test samples was randomly repeated 10 times, and the prediction results were averaged over the 10 trials. Classification results in Fig. 2C show that the classifier reliably predicted the location of astrocytes within the NAc with an overall accuracy of 78.66 ± 0.80%. The low SD indicates that the classifier performance was not significantly sensitive to the test sample selection. Classification accuracy in NAc subregions varied from 82.10 ± 1.62% for the AC to 74.43 ± 2.54% for the PC, suggesting that astrocytes were potentially more heterogeneous in the PC than in the AC (Fig. 2C).

To better understand the discriminative power of astrocyte morphometric features, they were ranked in order of importance using the mean decrease in accuracy (MDA), a method commonly used to estimate feature importance in decision tree–based ensemble models such as Random Forest. MDA estimated the impact of each feature on the model’s performance by measuring the average decrease in accuracy across all decision trees within the ensemble after removal of each feature. Figure 2D shows that sphericity was the most relevant feature for predicting astrocyte location, followed by fractal dimension and spherical disproportion. Both sphericity and spherical disproportion quantified the overall shape of the region occupied by a single astrocyte, while fractal dimension measured cell irregularity, including the complexity of elongated processes.

**Fig. 3.**
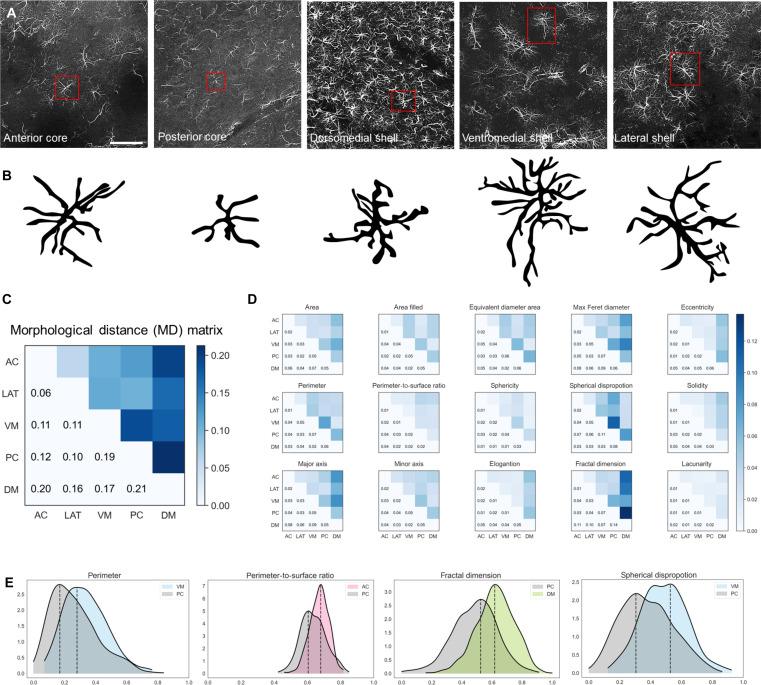
Astrocyte heterogeneity in the NAc. (**A** and **B**) Astrocytes in the NAc displayed significant morphological heterogeneity quantified using an appropriate notion of distance. Scale bar, 80 μm. (**C**) The MD matrix displays the pairwise distance in morphological space between the five NAc subdivisions, with a darker color indicating larger distances and greater morphological difference between the corresponding subregions. (**D**) MD was computed, aggregating the EMD between astrocytes in the NAc subdivisions for each of the 15 morphological features. (**E**) Empirical distributions of selected morphological features of astrocytes in different NAc subregions, with the dashed line indicating the median value of the feature (after normalizing the feature dynamic range in the interval [0.1]).

Predictive analysis results based on single-cell morphology confirmed the visually observed heterogeneity of NAc astrocytes (Fig. 3, A and B). To establish a measure of pairwise similarity/dissimilarity between astrocyte subpopulations across NAc subdivisions, we introduced a mathematical concept termed MD within feature space, which compared morphometric characteristics of astrocyte subpopulations and quantified their degree of shape likeness or variation. MD measured the combined dissimilarity over all measured morphometric features, where, for each feature, dissimilarity was measured using the well-established earth mover’s distance (EMD). We used MD to compute pairwise distances between astrocytes in different NAc subregions and reported the results in the MD matrix of Fig. 3C, where MD values are displayed both numerically and in color, with darker blue indicating larger distance and hence greater structural dissimilarity**.** The MD matrix shows that astrocyte structure in AC was most similar to astrocyte structure observed in LAT, followed by VM. Astrocyte structure in PC and DM regions and AC and DM regions was furthest apart from one another, with respect to MD. The matrix also shows that DM had the most distinct astrocyte morphology. Figure 3D reports the EMDs of each individual morphometric feature, and Fig. 3E compares the empirical distributions of some relevant shape features for additional interpretation of different properties quantified by the EMD. It is apparent that some feature distributions lacked symmetry, making the population mean difference a less reliable metric than EMD, which accurately captured disparities within feature space. The plots of the empirical distributions of perimeter and spherical disproportion in Fig. 3E show that the PC distributions were distinct from distributions in neighboring VM, with a lower median value for both features, suggesting that PC astrocytes were characterized by a rounder convex hull than VM astrocytes. The plots of the empirical distribution of the fractal dimension show that the distribution observed in the DM was rather distinct from that in the PC, with a higher median value, indicating richer astrocyte arborization. These observations are consistent with the EMD table of the fractal dimension, showing that astrocyte structure in DM was the most distinct from all other regions (Fig. 3D). The plot of the empirical distribution of the perimeter-to-surface ratio showed that astrocyte structure within the PC and AC regions was also distinct, with PC astrocytes having a lower median value, indicating a less complex shape than AC astrocytes.

The above observations about astrocyte heterogeneity within the NAc were consistent with findings from classical statistical analysis [analysis of variance (ANOVA)], showing that several morphometric features were statistically different between astrocyte subpopulations across NAc demarcations (table S1). For instance, most astrocyte features were highly significantly different statistically (*P* < 0.001) between VM and PC, DM and PC, and AC and PC but not between AC and LAT. While consistent, this statistical analysis is less specific and informative than the predictive single-cell analysis (Fig. 2) and the detailed geometric analysis (Fig. 3) discussed above.

To determine the utility of this approach across species and the generalizability of the astrocyte structural heterogeneity we observed across NAc subdivisions in rats, we performed a parallel study using NAc tissue from untreated male and female mice. We observed a similar degree of structural heterogeneity across NAc subdivisions in mice, with DM and VM emerging as regions where astrocyte structure was most distinct from that observed in other portions of the NAc (fig. S1).

### Astrocyte structure in the NAc predicted natural reward or drug use and drug seeking

**Fig. 4.**
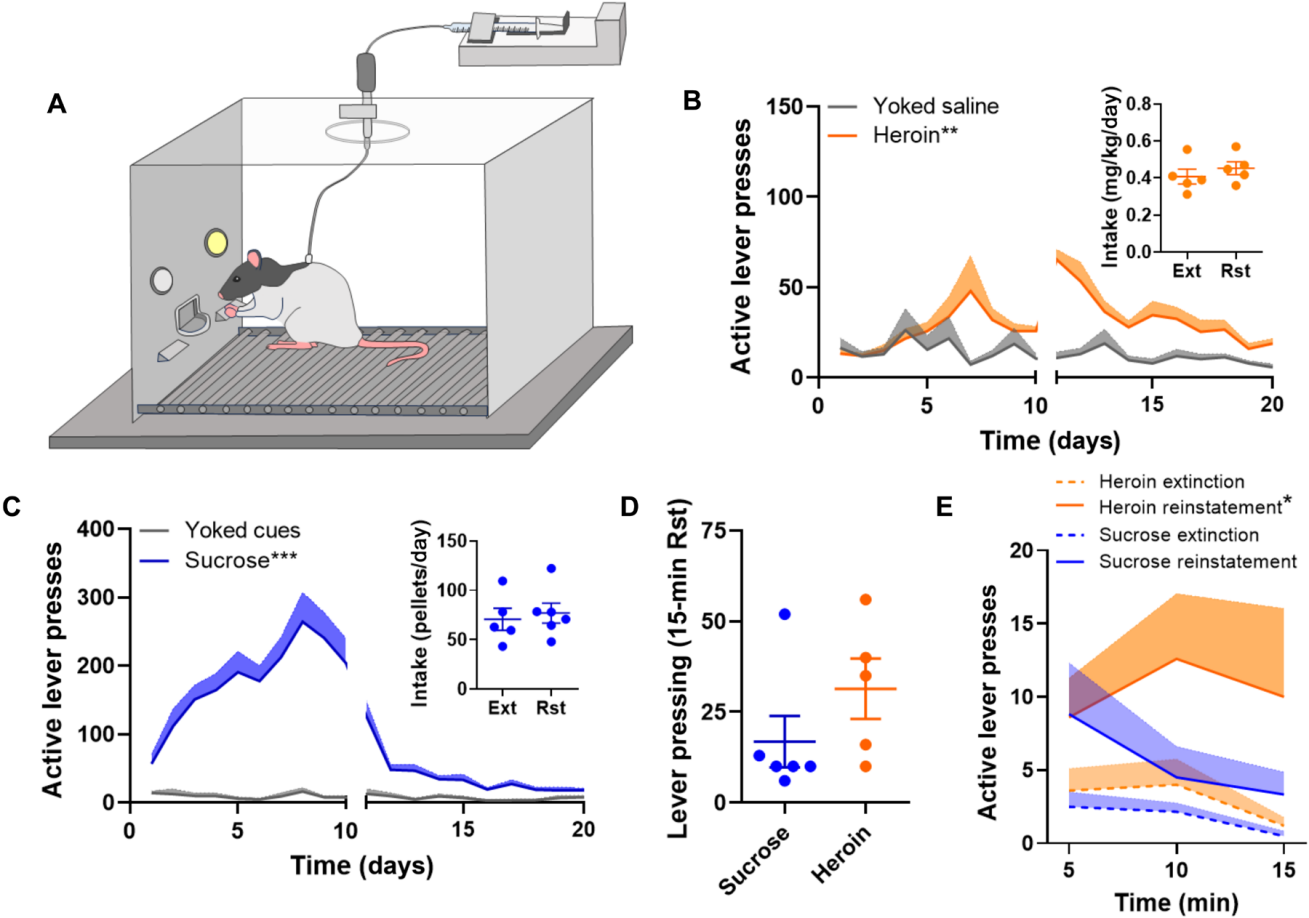
Rats that self-administered heroin exhibited perseverative drug seeking in the absence of reward. (**A**) Rats were implanted with intrajugular catheters and trained to self-administer heroin. (**B**) Active lever presses were paired with intravenous heroin infusions and light and tone cues during the 10 days of self-administration. Yoked saline animals received intravenous saline infusions when paired animals received heroin. Animals then underwent 10 days of extinction (Ext) training, where heroin and cues were withheld in response to active lever pressing. (**C**) Rats that self-administered oral sucrose pellets served as controls. (**D**) Exposure to light and tone cues was used to reinvigorate lever pressing in rats that had self-administered heroin or sucrose. (**E**) During reinstatement (Rst) of lever pressing, heroin-trained animals persisted in their lever pressing despite the absence of reward delivery compared with sucrose-trained animals. *P < 0.05, **P < 0.01, and ***P < 0.001 versus [(B) and (C)] yoked controls or (E) sucrose reinstatement.

We next investigated the hypothesis that astrocyte morphological characteristics from an individual animal were predictive of that animal’s history of heroin or sucrose self-administration and reward availability. Rats were trained to self-administer heroin by pressing an active lever, and heroin infusions were paired with light and tone cues (Fig. 4A); yoked control animals received saline infusions when paired animals self-administered heroin. Animals then underwent a withdrawal period where heroin was not delivered in response to active lever pressing (Fig. 4B). To compare the effects of natural reward versus drug use and drug seeking, rats in a parallel experiment received oral sucrose tablets instead of heroin infusions in response to active lever pressing (Fig. 4C). For a proportion of animals in both groups, reward seeking was reinstated for 15 min by exposure to compound cues previously paired with reward delivery (Fig. 4D). Rats that reinstated sucrose seeking extinguished lever pressing within 15 min in the absence of reward delivery compared with heroin-trained rats that continued to press the formerly active lever throughout the reinstatement session (Fig. 4E). Rats that underwent sucrose self-administration and then extinguished sucrose seeking or that extinguished and reinstated sucrose seeking are referred to as sucrose “withdrawal” and “relapse” herein for the sake of comparison with heroin-trained rats.

Multiclass Random Forest classifiers were trained on the collected single-cell morphometric features by randomly assigning 70% of the data for training and the remaining 30% for testing. As above, the data split was randomly repeated 10 times, and the prediction results were averaged over 10 trials to avoid potential bias in a single training or test sample assignment. We computed separate predictive models for each anatomical subdivision of the NAc for heroin or sucrose use, using three classification groups labeled as control, withdrawal, and relapse. Classification results showed that prediction accuracy values after heroin use ranged between 63 and 52% across NAc subdivisions. These results are shown in Table 1, where differences in counts of analyzed cells reflect differences in cell density between subregions and treatment conditions, since the same number of images and animals was analyzed for each NAc subdivision. These numbers were well above the 33% random chance of selecting one of the three classes correctly, indicating that drug experience affected astrocyte morphology. Nevertheless, classification accuracy was significantly below 100%, suggesting that an astrocyte group might respond to withdrawal, but not relapse, or that astrocyte plasticity may be heterogeneous within the same NAc subdivision. A more detailed analysis examining classification sensitivity and precision for withdrawal and relapse separately confirmed this conjecture (table S2). In the DM, for instance, the classification performance for heroin relapse had sensitivity = 61%, precision = 73%, and F1 score = 67%, while for withdrawal, we found sensitivity = 52%, precision = 57%, and F1 score = 54%. This suggests that astrocyte alterations in the DM were more prominent during relapse than during withdrawal. Similar observations applied after natural reward use, where accuracy was not only well above the 33% baseline of random chance but also well below 100%. Likewise, in this case, we observed very different classification sensitivity of AC and VM astrocytes to withdrawal and relapse (table S2). To determine whether sex differences in astrocyte structure contributed to the heterogeneity we observed, we computed predictive models separately for astrocytes from male and female rats for each NAc subdivision. We found significant prediction accuracy in both sexes, despite subtle differences in accuracy for each sex across subdivisions, with the most pronounced difference observed in the AC (table S3).

**Table 1.** Morphological alterations of NAc astrocytes predicted withdrawal and relapse. The table reports astrocyte cell count for each NAc location (column 1) and treatment (control, withdrawal, or relapse) for the heroin (columns 2, 3, and 4) and sucrose (columns 6, 7, and 8) experiments. It also reports treatment prediction accuracy (shown as means ± SD) using a Random Forest classifier, when the NAc location was fixed (columns 5 and 9, for the heroin and sucrose experiments, respectively).

NAc location	Heroin				Sucrose			
	Control	Withdrawal	Relapse	Accuracy	Control	Withdrawal	Relapse	Accuracy
AC	326	197	181	63.03 ± 0.01	60	66	31	47.28 ± 0.02
DM	1032	908	759	50.35 ± 0.01	100	101	142	62.03 ± 0.01
LAT	322	222	425	54.25 ± 0.00	95	122	60	53.30 ± 0.03
VM	281	214	231	52.30 ± 0.02	50	54	29	60.97 ± 0.02
PC	735	500	488	59.14 ± 0.01	111	93	75	45.94 ± 0.01

### Astrocyte morphology within the dorsomedial or ventromedial NAc shell predicted heroin or sucrose availability, respectively

To further assess the degree of morphological plasticity of NAc astrocytes in response to cues indicating heroin availability, we computed the MD of astrocyte subpopulations associated with withdrawal or relapse versus heroin-naïve controls. We computed this distance separately for each of the NAc subdivisions and repeated the same analysis for astrocytes from sucrose-trained animals. This analysis was used to quantify addiction-induced alterations in astrocyte morphology and identify NAc subregions that were most sensitive to reward seeking in opioid versus sucrose models.

**Fig. 5.**
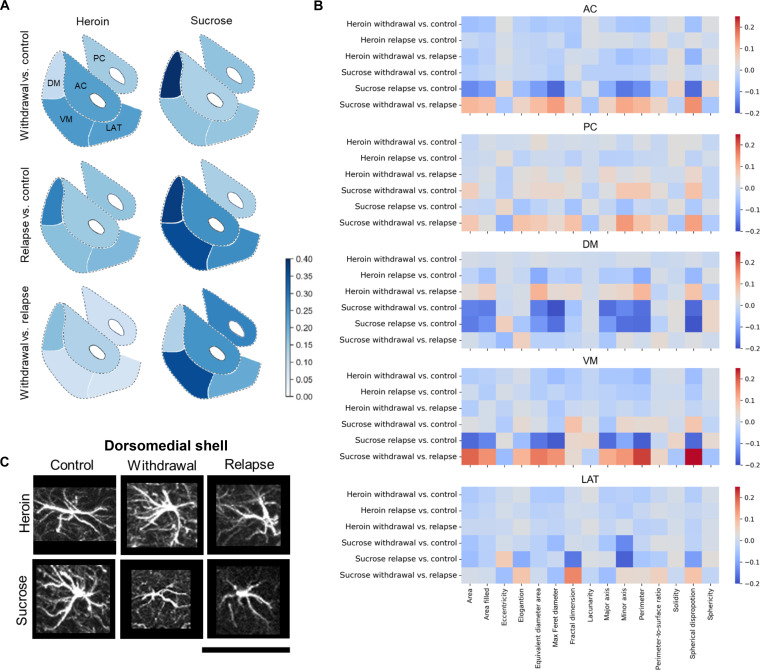
Morphological alterations of NAc astrocytes during withdrawal and relapse. (**A**) MD quantifies the level of heroin- or sucrose-induced cellular alterations during withdrawal and relapse in each one of NAc subregions (AC, LAT, VM, PC, and DM). Darker blue color indicates larger distance and greater morphological difference in withdrawal versus control, relapse versus control, and withdrawal versus relapse. (**B**) For each of the 15 morphological features (in column), the EMDs of withdrawal versus control, relapse versus control, and withdrawal versus relapse in the heroin and sucrose cases. Here, blue color indicates a positive change, and red color indicates a negative change. (**C**) Representative astrocyte images from DM subregion of the NAc exhibit different morphological characteristics in response to heroin or sucrose withdrawal and relapse. Scale bar, 50 μm.

Figure 5A illustrates the MD of astrocyte subpopulations in withdrawal versus control, relapse versus control, and withdrawal versus relapse conditions. In heroin withdrawal, astrocyte structure within the AC, VM, and LAT regions was most affected, while astrocyte structure within the DM was significantly less affected. By contrast, in comparisons of astrocyte structure during heroin relapse versus control and heroin relapse versus withdrawal, astrocyte structure within the DM region was the most affected, with astrocyte structure in all other subregions being significantly less altered. Instead, after extinction of sucrose self-administration, astrocyte structure was most altered within the DM, while during sucrose seeking, astrocyte structure was most affected in the VM. In sum, comparative analysis of opioid and sucrose models indicated a noticeably different response of DM and VM astrocytes to drug versus natural reward availability and seeking.

### Heroin use limited astrocyte structural plasticity

While MD quantified astrocyte morphological alteration overall, a closer inspection of the EMDs of individual morphometric features provided additional details of physical alterations observed. EMDs are reported in Fig. 5B as a heatmap, with blue indicating a decreased median and red indicating an increased median with respect to the reference group, i.e., red in withdrawal versus control indicates that the median value of the given feature has increased in withdrawal with respect to control, and blue in withdrawal versus relapse indicates that the median value of the given feature has decreased in withdrawal with respect to relapse.

Consistent with Fig. 5A, we observed that astrocyte structure in the DM was virtually unaffected during heroin withdrawal (Fig. 5B). After extinction of sucrose seeking, however, a large negative EMD was measured for max Feret diameter, major axis, and spherical disproportion in the same subregion. During heroin relapse, astrocyte structure in the DM showed significant negative EMD values for equivalent diameter area, perimeter, and spherical disproportion when compared with astrocytes from control animals. In opioid withdrawal versus relapse, the EMDs of the same features were negative, confirming that after heroin, DM alterations measured during relapse were absent during withdrawal. The EMD values of the abovementioned features were relatively unchanged during sucrose seeking. Figure 5C shows representative images from the DM. In accord with the observations reported above, astrocytes appeared significantly altered in opioid relapse where they were smaller and with reduced processes; however, no such alterations were visible in withdrawal. By contrast, after sucrose use, astrocytes were visibly altered at both withdrawal and relapse time points.

Unlike heroin, during sucrose seeking, the most notable changes occurred in the VM, where EMD values of several astrocyte features were prominently altered with negative values including area, max Feret diameter, major axis, perimeter, and spherical disproportion (Fig. 5B). After sucrose extinction, the same features had minimal alterations, in some cases with the opposite value. The sucrose withdrawal versus relapse comparison showed that the EMDs of the same features were positive, confirming that alterations in astrocyte structure in the VM measured during sucrose seeking were absent in withdrawal. After opioid withdrawal, significant negative EMD values were measured for max Feret diameter, major axis, perimeter, and spherical disproportion in the VM. During heroin relapse, the EMDs of the same features were lower, with reduced values. This is confirmed in the withdrawal versus relapse map, where the EMD values of the same features were positive, showing that after heroin, the same VM alterations found in withdrawal were present, albeit reduced, in relapse.

In the other regions, EMD values were less prominent. During sucrose seeking, patterns of astrocyte structural plasticity observed in the AC and LAT were similar to what was observed in the VM but at a reduced scale. Namely, the EMD values of several features were altered with a reduced value, but the alterations measured in relapse were absent in withdrawal. Similarly, during heroin withdrawal, astrocyte structural changes in the AC and LAT were similar to what we observed in the VM albeit reduced. This suggests that astrocytes in AC and LAT underwent a similar pattern of morphological plasticity to VM astrocytes.

The above observations about astrocyte structural alterations in response to heroin or sucrose availability were consistent with ANOVA, showing that several morphometric features were significantly different between control and withdrawal, control and relapse, or withdrawal and relapse in specific NAc subdivisions (table S4). For instance, most mean features in DM exhibited highly significant statistical difference (*P* < 0.001) between control and heroin relapse but not between control and heroin withdrawal. By contrast, most features in DM exhibited highly significant statistical difference between control and sucrose withdrawal and between control and sucrose relapse. While this statistical analysis is consistent with our observations above, it is less informative than the predictive single-cell analysis and the detailed geometric analysis discussed in Fig. 5.

To compare our findings on astrocyte cytoskeletal plasticity using GFAP as a marker with those assessing motility of finer perisynaptic astrocyte processes, we labeled NAc astroglia from heroin-trained rats with a virally expressed membrane-bound reporter (*29*). Despite no overlap between the GFAP cytoskeleton and the finest peripheral processes labeled using a membrane reporter (fig. S2A), we observed significant differences in astrocyte-synapse association across NAc subdivisions, with the largest degree of astrocyte-synapse adjacency in VM in control animals (fig. S2B). We also found significant reductions in astrocyte structural complexity after heroin use across NAc subdivisions (fig. S2C), consistent with observations made using GFAP as a cytoskeletal marker (Fig. 5B). While astrocyte-synapse association was recovered in PC during relapse, significant retraction of perisynaptic processes remained during relapse in VM and LAT, NAc subdivisions associated with extinction of conditioned responses (fig. S2C).

## DISCUSSION

Astrocyte heterogeneity in structure and function is an emerging area of research interest, and, during the past few years, a small number of numerical algorithms for astrocyte detection and clustering or classification were introduced to facilitate this effort (*38*–*41*). However, relatively little is known about how to assess and interpret astrocyte morphology on a broad scale, highlighting the need for new objective approaches to identify and quantify astrocyte structural heterogeneity across brain regions and in disease conditions. In response to this need, we developed an automated pipeline for the accurate extraction and rigorous quantification of single-cell morphological features of individual astrocytes in the brain, and we demonstrate its utility in multiple rodent species, including both mice and rats. Our pipeline uses state-of-the-art supervised machine learning for detection and segmentation of individual astrocytes and highly sensitive geometric tools to quantify alterations in morphology. We report the results of several numerical experiments conducted to find, detect, and analyze astrocyte subpopulations in the NAc using single-cell morphological characteristics. Our results include a number of fundamental findings: That single-astrocyte morphological characteristics are predictive of astrocyte location within the NAc; that single-astrocyte morphological characteristics are predictive of reward availability; and that single-astrocyte morphological alterations are highly specific, in the sense that alterations observed during heroin seeking are distinct from those observed during natural reward seeking. We found notable heterogeneity of astrocyte structure across the NAc in both male and female animals that predicted cell location across NAc subdivisions that we define here but that are not formally recognized based on classic molecular or anatomical markers. For example, the NAc is classically subdivided into core and shell subregions but is not frequently subdivided beyond these designations. Our findings confirm that there is not a standard astrocyte type that exists across the brain or even portions of the same structure, i.e., ventral striatum, but that astrocyte structure may be distinct across adjacent and functionally similar brain regions. That astrocyte structure is unique across NAc subdivisions, i.e., AC versus PC and DM versus VM versus LAT, indicates potential unrecognized or underappreciated differences in signaling within these subdivisions that may recruit the unique forms of astrocyte structural plasticity observed here.

We used heroin self-administration, extinction, and reinstatement in rats to model opioid use disorder, withdrawal, and relapse and compared astrocyte morphological plasticity across these time points in animals trained to self-administer and seek the natural reward sucrose. We found that structural alterations in NAc astrocytes predicted an animal’s exposure to heroin or sucrose, as well as an animal’s behavioral response to each reward, i.e., withdrawal or seeking. It is notable that heroin and sucrose experience were encoded by astrocyte structure differently across NAc subdivisions, with astrocyte structure within the VM portion of the NAc shell uniquely affected during sucrose seeking and astrocyte structure within the DM portion of the NAc shell uniquely altered during heroin relapse. Astrocytes in the NAc are critically involved in regulating neural responses to drug cues that drive seeking but have not previously been shown to be engaged during seeking of natural rewards such as food or sucrose. Our findings further confirm previous observations that the AC harbors drug-related but not natural reward–related plasticity (*33*). The data presented here demonstrate that astrocytes are sensitive to the presence of natural rewards and that astrocyte plasticity is engaged in nonoverlapping portions of the NAc when an animal seeks a drug versus a nonpathological reinforcer.

Last, our results indicate that astrocyte structural plasticity is more dynamic in sucrose-trained compared with heroin-trained animals, with a loss of astrocyte structural plasticity notable in heroin-exposed animals in all NAc subregions aside from the DM. Our own previous work has shown that astrocyte morphological plasticity in the NAc and other basal ganglia structures serves to dampen drug seeking (*29*–*31*). Together, our findings suggest that self-administration of sucrose as a reinforcer compared with heroin permits better engagement of astrocyte plasticity during extinction training and may enable reversal learning required to dampen seeking in the absence of reward. The most notable plasticity observed in astrocytes from heroin-trained animals occurred in the DM portion of the NAc shell during seeking, whereas notable plasticity was observed across portions of the NAc shell, including the VM after sucrose use and during sucrose seeking. Data from others support a role for various portions of the NAc shell, including the VM, in the extinction of conditioned responding when a reinforcer is removed. For instance, projections from the VTA to the VM are activated by unexpected aversive outcomes, including the absence of anticipated reward (*42*), and excitatory projections from the infralimbic cortex to the VM are recruited during extinction training (*43*, *44*). That astrocyte plasticity was engaged more effectively after use of a nonpathological reinforcer in portions of the NAc associated with extinction of conditioned responses is consistent with less perseverative reward seeking following food or sucrose self-administration compared with chronic drug self-administration (Fig. 4E).

Astrocytes are positioned adjacent to most of the excitatory synapses in the brain, where they take up glutamate during synaptic activity, and signal with pre- and postsynaptic elements to regulate synaptic activity and plasticity (*3*). Given the dense and functionally important excitatory inputs to the NAc that regulate drug seeking and its extinction, NAc astrocytes are situated to regulate signaling involved in drug use and relapse (*14*, *29*, *30*, *45*–*47*). Despite no overlap between the astrocyte cytoskeleton labeled with GFAP and the finest peripheral processes, we found notable similarities in results obtained using our newly developed pipeline and previously validated approaches that detect astrocyte fine process motility (*29*). Consistent with previous observations made using these techniques, our data show that astrocytes undergo vast structural rearrangements following drug use, and we predict that these structural changes likely reflect changes in astrocyte function. We previously showed that opioid use is associated with reduced GFAP expression in the NAc (*41*), and this reduction may contribute to the reduced astrocyte structural plasticity that we observed after heroin use in portions of the NAc associated with extinction learning. Whether and how GFAP down-regulation also contributes to the widespread reductions in astrocyte-synapse adjacency that we observe across the NAc after heroin use is an open question. To the extent that type III intermediate filament proteins such as GFAP serve as scaffolding for intracellular protein trafficking, GFAP structure may be an indicator of astrocyte function and may affect the trafficking of proteins involved in fine process dynamics and neurotransmitter uptake (*48*–*50*). Decreases in GFAP and ezrin, as well as important astroglial surface proteins involved in glutamate regulation (i.e., xCT and GLT-1) in animal models of substance use disorders, support this hypothesis (*29*, *41*, *51*, *52*).

The analysis presented in this paper relies, in part, on the accuracy of the astrocyte detection and segmentation process. Automated detection and segmentation of astrocytes in micrographs remain highly challenging, with deep learning methods—such as the approach used in this study—offering state-of-the-art performance. While these methods achieve accuracy comparable to that of domain experts in identifying individual astrocytes, they inherently reflect the limitations and potential biases of the expert annotators, whose annotations were used to train the model. For further discussion on the development and limitation of these deep learning models, please refer to (*41*). Further, while GFAP is the most commonly used marker for assessment of astrocyte structure across species, not all astrocytes express GFAP in either rodents or humans (*53*). Alternative approaches, including current vector–based methods that provide valuable information regarding astrocyte-circuit interactions in rodents (*30*, *37*, *54*), are not feasible in postmortem human tissue, limiting what can be known about how findings in animal models translate to human disease. Thus, a significant strength of our approach is that it can be feasibly applied across species, including to images generated from humans for comparison with animal models of disease. Efforts are ongoing to understand the biological significance of GFAP expression, or lack thereof, in astrocyte subpopulations and to identify additional cytoskeletal markers that may be expressed in astrocytes lacking GFAP. Should better markers of astrocyte cytoskeletal structure become available, our deep learning pipeline will facilitate analysis of astrocyte heterogeneity and plasticity using newly identified markers.

## MATERIALS AND METHODS

### Ethics declaration for animal experiments

Animal procedures and experiments were in line with standards and regulations to reduce the suffering of the animals approved by the Institutional Animal Care and Use Committee at the University of Cincinnati (UC). UC complies with the US Department of Agriculture Animal Welfare Act (Public Law 89-544), as amended by PL91-579 (1970), PL94-279 (1976), and 45 CFR37618 (1980), and Health Research Extension Act of 1985 (Public Law 99-158) and follows the Public Health Service Policy on Humane Care and Use of Laboratory Animals (revised, September 1986), the Guide for the Care and Use of Laboratory Animals (revised, September 1986), and the Guide for the Care and Use of Laboratory Animals of the Department of Health, Education, and Welfare 85-23 (revised, 1985). All authors complied with the Animal Research Reporting of In Vivo Experiments guidelines.

### Self-administration

Adult male and female Long-Evans rats were trained to self-administer oral sucrose pellets or intravenous heroin infusions via an implanted intrajugular catheter over 10 days (*29*, *30*). During self-administration, active lever presses were paired with oral sucrose (45 mg) or intravenous heroin delivery (100 μg per infusion on days 1 and 2, 50 μg per infusion on days 3 and 4, and 25 μg per infusion on days 5 to 10) and light and tone cues for 5 s. Yoked control animals were exposed to light and tone cues when a paired animal received sucrose or heroin, and rats yoked to heroin self-administering animals also received intravenous saline infusions. After self-administration, rats underwent 10 to 12 days of extinction training, where cues and reward delivery were withheld in response to active lever pressing. Animals in yoked control and extinction groups were euthanized 24 hours after the last extinction session, and their brains were extracted for analysis. Remaining animals underwent a 15-min reinstatement session where cues were restored to active lever presses, but no rewards were delivered, before euthanasia and tissue processing. At the start of the reinstatement session, animals were exposed to one experimenter-administered compound cue to initiate lever pressing.

### Intracranial microinjections (*29*)

While under anesthesia for jugular catheter implantation, rats received infusions of AAV5-GfaABC1D-Lck-GFP (Addgene) in the NAc [+1.5 mm anteroposterior (AP), ±1.7 mm mediolateral (ML), −7.0 mm dorsoventral (DV)]. Sufficient virus was delivered (1.5 μl per hemisphere, 0.15 μl/min, and 5-min diffusion) such that astrocyte labeling was detectable in all NAc subdivisions investigated here (AC, PC, DM, VM, and LAT). Viral incubation occurred over the course of operant conditioning.

### Tissue preparation and image acquisition (*41*)

After completion of behavioral protocols, 31 rats (13 females and 18 males) and 5 untreated mice (3 males and 2 females) were perfused transcardially with 4% paraformaldehyde, and brains were incubated in 4% paraformaldehyde overnight at 4°C. Tissue was sliced coronally at 50 μm using a vibrating blade microtome (Leica Microsystems), a thickness likely to capture most of the GFAP branches of astrocytes positioned near the middle of the tissue and contain few stray branches from cells from above and below (table S5). Tissue slices were stored in glycerol-based media at 4°C until staining. Slices containing the NAc were incubated in 1× phosphate-buffered saline (PBS) with 2% Triton X-100 for 15 min at room temperature before incubation in PBS with 0.2% Triton X-100 with 2% normal goat serum (block) for 1 hour at room temperature shaking gently. Slices were then incubated in primary antibody (chicken anti-GFAP, Abcam, ab4674 or rabbit anti–synapsin I, Abcam, ab64581) at 1:1000 in block for 24 hours at 4°C shaking gently. Tissue was rinsed in PBST before overnight incubation in fluorescently conjugated secondary antibody (Alexa Fluor) in PBST at room temperature. After rinsing, tissue was mounted onto glass slides using ProLong Gold Antifade Reagent and imaged using a Leica Stellaris 5 confocal microscope using a 20× objective lens. An average of 40 16-bit images was collected per stack at 600 Hz with a frame size of 1024 × 1024 and a 0.5-μm step size, for a final image size of 579.44 μm^2^ and a pixel size of 566.4 nm^2^. Images were collected from five different subregions of the NAc including the AC, PC, DM, VM, and LAT by comparison to (*55*). Acquired image stacks were converted into planar images by maximum projection before applying our astrocyte detection algorithm. Two hundred ninety-one stacks containing 5400 GFAP-labeled cells from sucrose self-administering animals, heroin self-administering animals, or controls were included in the present analysis. GFAP-labeled images from heroin self-administering animals were originally released in (*41*). Cell locations were manually annotated by domain experts without prior knowledge of detection results. To annotate images, we used Visual Geometry Group (VGG) Image Annotation, an open-source manual annotation software that allows the user to manually draw a rectangular box around an object in an image and save the corresponding coordinates (*56*). Images in this dataset have different sizes: 987 × 987, 774 × 774, 1014 × 1014, and 1058 × 1058 pixels. To assess astrocyte-synapse coregistration using virally expressed green fluorescent protein (GFP), fixed tissue slices immunolabeled for synapsin I were imaged using a 63× oil immersion lens. After image collection, 459 individual astroglia were analyzed for membrane coregistration with immunolabeled synapsin I using Bitplane Imaris (*29*), and the total volume of synapsin I labeling that coregistered with the astroglial membrane was normalized to astrocyte volume to obtain an index of astrocyte-synapse coregistration for each cell analyzed. All imaging was conducted by investigators blind to animal treatment and sex.

### Astrocyte processing pipeline

The pipeline illustrated in Fig. 1A consists of four processing units, and the implementation of the first two steps adapted deep learning algorithms developed by some of the authors (*40*, *41*). Details about each processing unit are summarized below.

1) Astrocyte detection was implemented using YOLOv5 (*57*), a deep learning platform for object detection that was trained using a large library of astrocyte images manually annotated by domain experts and optimized for the task of detecting GFAP-labeled astrocytes. This unit accepts input images with different sizes in pixels and outputs the predicted locations of astrocytes in the form of rectangular boxes adapted to cell location and size (Fig. 1B). We have shown in prior work that this approach yields state-of-the-art detection performance (precision = 0.81 to 0.86 and recall = 0.75 to 0.87 on different datasets tested) and has high throughput (time = 0.03 s to process an image of size 1024 × 1024 pixels) (*41*). As shown in Fig. 1B, the algorithm performs competitively in images where astrocyte density is relatively high, although in regions of the highest cell density, some astrocytes may be missed.

2) Astrocyte segmentation was implemented using a UNet-VGG16 deep learning architecture adapted from our prior work (*40*). This network concatenated the classical UNet architecture (*58*), which has been shown to be very effective for cell segmentation, with a pretrained VGG16 network (*59*) for increased performance. To reduce the number of training samples needed to generate a high-performing segmentation model, a training strategy was applied that included specially designed multiscale filters and a sparsity constraint (*40*). This approach was shown to yield state-of-the-art astrocyte segmentation performance (precision = 0.86, recall = 0.69, and F1 score = 0.75 on a similar astrocyte dataset) with efficient computing time (1 s to process one image) (*40*).

3) The feature extraction unit was used to compute single-cell morphometric characteristics from the segmented images of GFAP-labeled astrocytes (Fig. 1B). We selected 15 features, shown in Fig. 2A, that include area, area filled, equivalent diameter area, max Feret diameter, eccentricity, perimeter, perimeter-to-surface ratio, sphericity, spherical disproportion, solidity, major axis length, minor axis length, elongation, fractal dimension, and lacunarity. Although measures such as major axis length may be affected by astrocyte orientation relative to the imaging plane, measures involving ratios, including eccentricity, fractal dimension, and perimeter-to-surface ratio, are likely to be more robust to projection onto a plane. Further, some features, e.g., fractal dimension, have been used in the literature as interpretable descriptors of astrocyte geometry (*60*). We used the implementations available in the Python library scikit-image (*61*) except for the fractal dimension feature, for which implementation was taken from the PoreSpy library (*62*).

4) Morphological and statistical analysis used single-cell features computed above to assess astrocyte spatial heterogeneity and morphological alterations induced by addiction-related behaviors. We carried out two levels of analysis: (i) single-cell classification based on supervised learning and (ii) unsupervised quantification of similarities/dissimilarities between astrocyte subpopulations using a newly defined notion of distance in feature space. Significance and implementation details are described below.

i. Classification was applied to test whether single-cell morphological characteristics of an individual astrocyte could predict either the anatomical location within the NAc at baseline (AC, DM, LAT, VM, and PC) or addiction-related behavior (control, withdrawal, and relapse). For this predictive task, we applied Random Forest, a supervised probabilistic classifier that we trained using the 15 single-cell morphological features computed from the astrocyte images and listed above. To run this multiclass classification task with Random Forest, we used the “one versus rest classifier” strategy, where a binary classifier was trained for each class separately. Because the number of cells in each group associated with either anatomical region or addiction-related behavior was different, we applied the SMOTE algorithm (*63*) to balance the dataset. This method generated new sample points that were distributed closer to the center of the minority sample with a higher probability to avoid marginalization of the expanded data. For our experiments, we set the parameter balance ratio to 0.9, with SD in the interval (0, σ_0_/3), where σ_0_ was the SD of the data.

ii. MD definition was based on the well-established EMD, which is closely associated with the Wasserstein distance. It is designed to quantify dissimilarity between two empirical probability distributions by measuring the “minimal work” needed, based on an optimal transport framework, to transform one distribution into the other. Given any pair of astrocyte subpopulations, for each shape feature, we calculate the EMD between the empirical distributions or histograms of the corresponding morphometric feature. The MD aggregates the contributions of the different features while controlling their correlations. More precisely, let *d*[*P_m_*(*k*), *P_n_*(*k*)] denote the EMD between the empirical distributions of shape feature *k* in two astrocyte subpopulations indexed by *m* and *n*, and let *d*(*P_m_*, *P_n_*) be the column vector with components *d*[*P_m_*(*k*), *P_n_*(*k*)], where *k* = 1, …, *K*; we then defined the square of the MD between the astrocyte subpopulations indexed by *m* and *n* as

$$\begin{array}{c} MD^{2}\left(m,n\right)=d^{t}\left(P_{m},P_{n}\right)\;R\;d\left(P_{m},P_{n}\right)\\ -\frac{1}{2I}d^{t}\left(P_{m},P_{n}\right)\;\text{abs}\left(R-I\right)\;d\left(P_{m},P_{n}\right) \end{array}$$

where *R* is the *K* × *K* Spearman correlation matrix of the *K* computed morphometric features, and *I* is the *K* × *K* identity matrix. Note that if the features are fully decorrelated, then *R* = *I* and, in this case

$$MD^{2}\left(m,n\right)=d^{t}\left(P_{m},P_{n}\right)\;R\;d\left(P_{m},P_{n}\right)=\sum_{\left\{k=1\right\}}^{K}d^{2}\left[P_{m}\left(k\right),P_{n}\left(k\right)\right]$$

so that all features contribute equally. However, if features were correlated, then the negative term would reduce the contribution of the correlated features in proportion to their correlation. By construction, MD is always nonnegative.

### Evaluation metrics

To assess classification performance, we used the standard binary classification metrics defined next. Sensitivity [or true positive (TP) rate or recall] measures the proportion of correctly classified cells with respect to the total number of cells in that class. Denoted by TP, the number of correct classifications, and by FN (false negative), the number of missed classifications, we define sensitivity *S* as

$$S=\frac{\text{TP}}{\text{TP}+\text{FN}}$$

Precision measures the proportion of correctly classified cells over all cells in that class. That is, denoted as FP (false positive), the number of wrongly classified cells, the precision *P* is

$$P=\frac{\text{TP}}{\text{TP}+\text{FP}}$$

Last, the F1 score is a measure of the overall effectiveness of the classification algorithm and is computed as

$$F1=2\;\frac{P*S}{P+S}=\frac{2*\text{TP}}{2*\text{TP}+\text{FP}+\text{FN}}$$

The F1 score ranges between 0 and 1, with F1 = 1 describing perfect classification.

Accuracy measures the proportion of all the correctly identified cases, and it is the most used performance metric when all the classes are equally important

$$\text{Accuracy}=\frac{\text{TP}+\text{TN}}{\text{TP}+\text{FP}+\text{TN}+\text{FN}}$$

### Statistical analysis

Standard statistical inference was applied using the following two-step procedure. First, a one-way ANOVA was conducted to test whether statistically significant differences existed between astrocyte subpopulations based on their morphological features. Second, the rejection of the null hypothesis in the one-way ANOVA resulted in performing pairwise tests with Tukey’s post hoc test method to test the difference between each pair of astrocyte subpopulations (tables S1 and S3). Data in fig. S2 were non-Gaussian, and statistical significance was calculated using Kruskal-Wallis, followed by Dunn’s multiple comparisons test.
