# Supplementary material for: Supervised and unsupervised learning reveal heroin-induced impairments in astrocyte structural plasticity
Source: Sci Adv. 2025 Apr 30;11(18):eads6841. doi: 10.1126/sciadv.ads6841 (PMC12042888; doi:10.1126/sciadv.ads6841)
Supplement: Supplementary file 1 — Figs. S1 and S2 Tables S1 to S5 [file sciadv.ads6841_sm.pdf]

Science Advances
AAAS

Supplementary Materials for

Supervised and unsupervised learning reveal heroin-induced impairments in astrocyte structural plasticity

Michela Marini *et al.*

Corresponding author: Anna Kruyer, kruyeraa@uc.edu; Demetrio Labate, dlabate@math.uh.edu

Sci. Adv. **11**, eads6841 (2025)
DOI: 10.1126/sciadv.ads6841

This PDF file includes:

Figs. S1 and S2
Tables S1 to S5

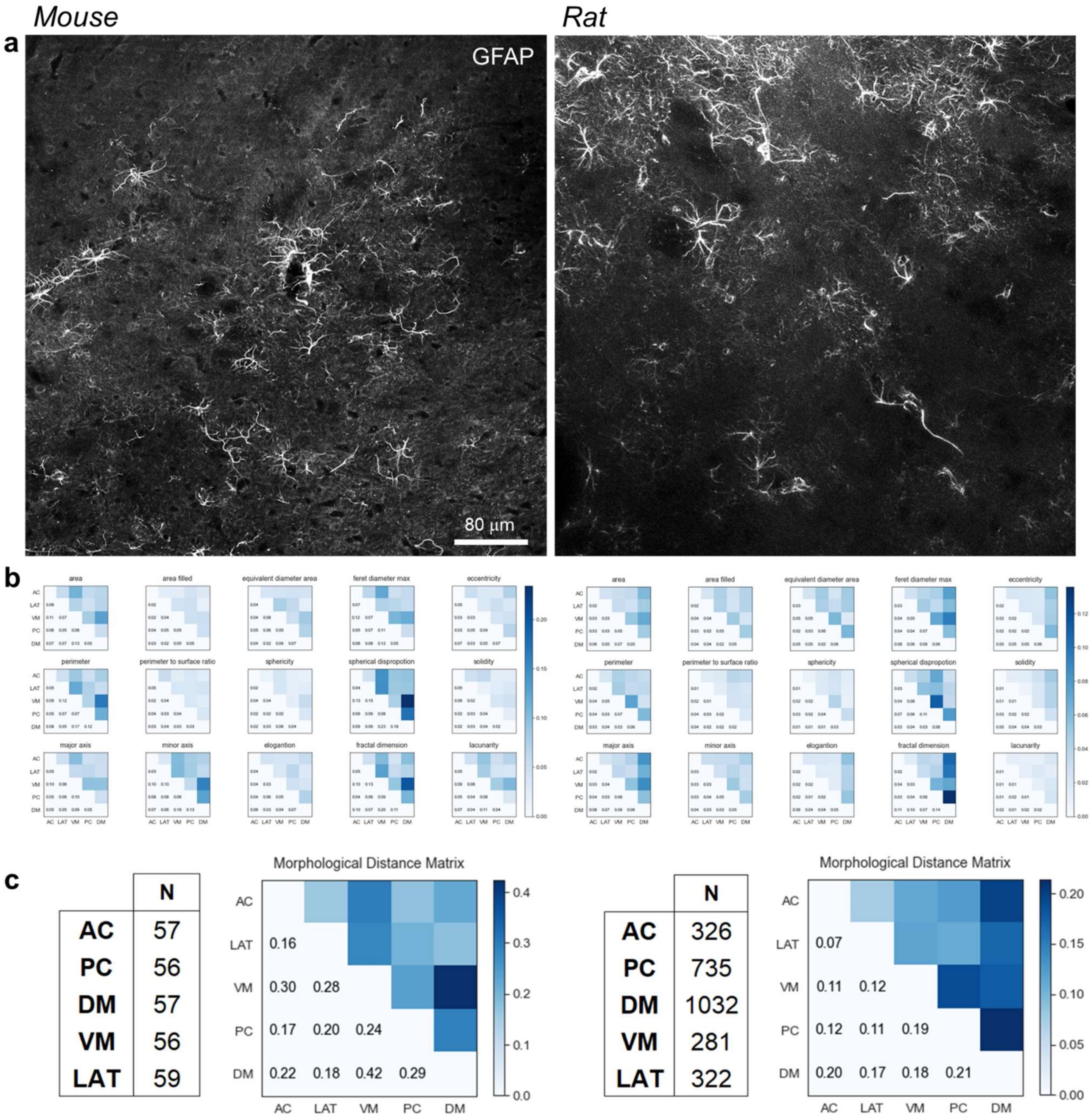

figure S1. Astrocyte structural heterogeneity across NAc subdivisions was observed in both mice and rats. Analysis of GFAP immunolabeling in mice and rats (LAT, **a**) revealed similar astrocyte structural heterogeneity across portions of the NAc (**b**). (**c**) Astrocyte structure was most distinct in DM of rats and in DM and VM of mice. Number (N) of analyzed cells shown in (**c**) along with MD matrices showing pairwise distance in morphological space between astrocytes across NAc subdivisions.

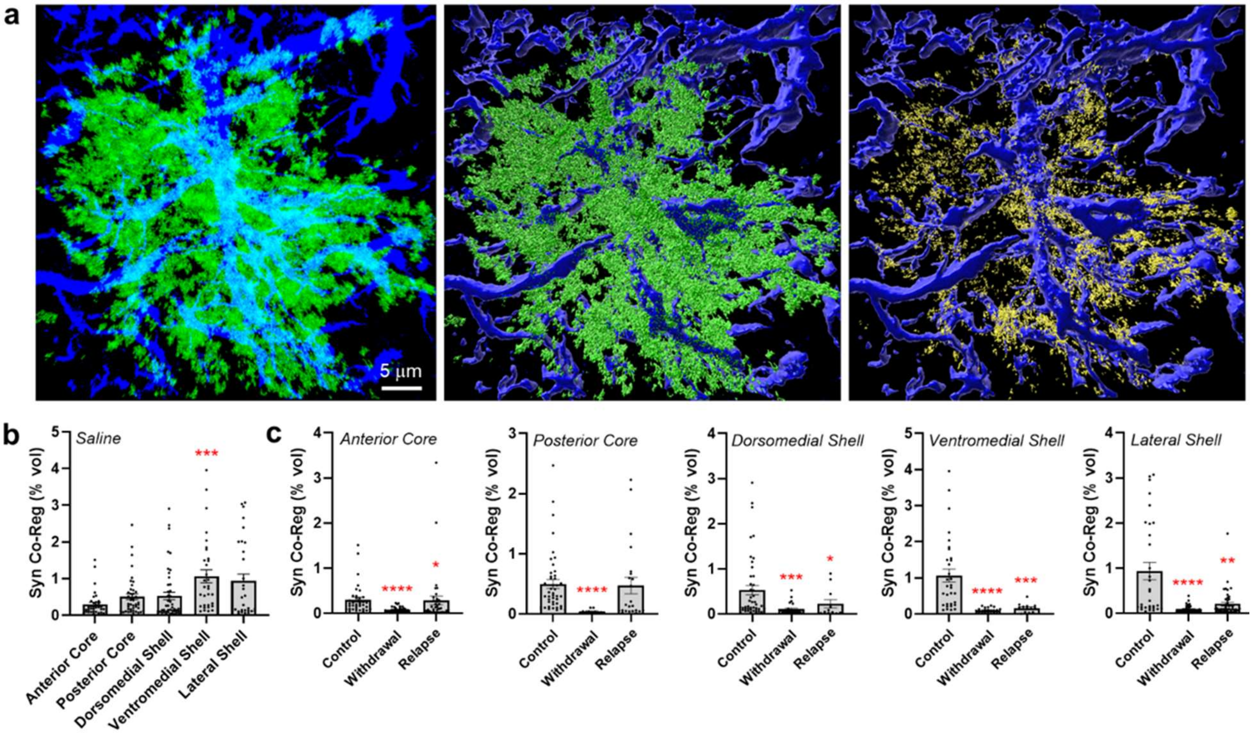

figure S2. Perisynaptic astrocyte processes were reduced by heroin use in all NAc subdivisions. (**a**) NAc astroglia (green) were labeled with membrane-bound GFP using AAV5-GfaABC1D-Lck-GFP (left panel). The relationship between the astrocyte membrane (green) or perisynaptic processes immunoreactive for the actin-binding protein ezrin (yellow) and the GFAP cytoskeleton (blue) is shown in a 3D render in (**a**, middle and right panels, respectively). (**b**-**c**) Co-registration of the astroglial membrane with immunoreactive Synapsin I puncta was quantified as in (*29*) to determine whether changes in GFAP structure observed after heroin use were reflective of fine process motility. Synaptic association of astrocyte processes was most prominent in the VM in control animals. (**c**) Withdrawal from heroin use caused retraction of perisynaptic astrocyte processes in all NAc subdivisions. Cue-induced relapse caused reinsertion of perisynaptic astrocyte processes in the PC, but not in other NAc subdivisions involved in extinction learning like the VM or LAT. Data in (**c**) were collected from AC: 36/6, 35/6, 39/7; PC: 45/7, 11/3, 23/5; DM: 46/3, 26/3, 12/2; VM: 32/5, 21/2, 14/3; and LAT: 31/5, 41/6, 47/5 cells/animals for Control (Saline), Withdrawal and Relapse, respectively. *$p<0.05$, **$p<0.01$, ***$p<0.001$, ****$p<0.0001$ vs. Control using Kruskal-Wallis followed by Dunn’s post-hoc test.

a

	ANOVA		NAc Location									
	F value	p value	AC-DM	AC-LAT	AC-VM	AC-PC	DM-LAT	DM-VM	DM-PC	LAT-VM	LAT-PC	VM-PC
area	26.693	1.008e-21	0	0.477	0.725	0.002	0	0	0	0.039	0.327	0
area filled	17.183	6.441e-14	0.001	0.999	0.001	0.799	0.001	0.972	0	0.003	0.659	0
equivalent diameter area	21.260	2.874e-17	0	0.993	0.001	0.541	0.001	0.996	0	0.004	0.252	0
feret diameter max	32.720	1.212e-26	0	0.545	0.521	0	0	0	0.001	0.021	0.064	0
eccentricity	23.377	5.254e-19	0	0.913	0.983	0.968	0	0	0	0.999	0.470	0.728
perimeter	21.561	1.627e-17	0.131	0.999	0.009	0.002	0.057	0.382	0	0.004	0.006	0
perimeter to surface ratio	29.647	3.874e-24	0	0.060	0.118	0	0	0	1	0.999	0	0
sphericity	48.004	5.604e-39	0.464	0.921	0.007	0	0.056	0.083	0	0	0	0
spherical disproportion	37.298	2.324e-30	0.945	0.818	0.020	0	0.227	0.023	0	0	0	0
solidity	74.012	1.955e-59	0	0.475	0.532	0	0	0	0	1	0	0
major axis	35.825	3.627e-29	0	0.491	0.997	0	0	0	0	0.326	0.067	0
minor axis	6.790	1.963e-05	0.968	1	0.810	0.008	0.978	0.299	0.002	0.787	0.010	0
elogantion	27.669	1.600e-22	0	0.582	0.770	1	0	0	0	0.999	0.475	0.710
fractal dimension	122.717	6.235e-96	0	0.919	0.008	0.016	0	0	0	0.086	0	0
lacunarity	20.413	1.427e-16	0	0.157	1	0.999	0.025	0	0	0.272	0.032	0.991

b

	NAc Location									
	AC-DM	AC-LAT	AC-VM	AC-PC	DM-LAT	DM-VM	DM-PC	LAT-VM	LAT-PC	VM-PC
area	***			**	***	***	***	*		***
area filled	***		**		***		***	**		***
equivalent diameter area	***		***		***		***	**		***
feret diameter max	***			***	***	***	***	*		***
eccentricity	***				***	***	***			
perimeter			**	**			***	**	**	***
perimeter to surface ratio	***			***	***	***			***	***
sphericity			**	***			***	***	***	***
spherical disproportion			*	***		*	***	***	***	***
solidity	***			***	***	***	***		***	***
major axis	***			***	***	***	***			***
minor axis				**			**		**	***
elongation	***				***	***	***			
fractal dimension	***		**	*	***	***	***		***	***
lacunarity	***				*	***	***		*	

table S1. Morphometric characteristics across demarcations of NAc are statistically different. (**a**) F- and p-values of one-way ANOVA and p-values of the corresponding pairwise tests (with the Tukey's HSD method) to compare the difference between the means of a given feature across the five NAc demarcations. Null hypothesis states that the means are equal among the five NAc demarcations for each feature. ANOVA test has 4 degrees of freedom between groups and 2562 degrees of freedom within groups. (**b**) p-values using Tukey's HSD pairwise tests in (**a**). *p<0.05, **p<0.01, ***p<0.001.

Heroin

	S	P	F1
**AC**			
control	78.49	61.34	68.87
withdrawal	61.29	73.08	66.67
relapse	70.65	80.25	75.14
**DM**			
control	58.90	47.03	52.30
withdrawal	52.10	56.69	54.30
relapse	60.84	73.44	66.55
**LAT**			
control	55.34	51.82	53.52
withdrawal	61.17	68.48	64.62
relapse	50.49	48.60	49.52
**VM**			
control	52.50	46.67	49.41
withdrawal	53.75	51.19	52.44
relapse	46.91	56.72	51.35
**PC**			
control	59.18	50.43	54.46
withdrawal	61.93	60.70	61.31
relapse	58.16	72.15	64.41

Sucrose

	S	P	F1
**AC**			
control	55.00	44.00	48.89
withdrawal	40.00	50.00	44.44
relapse	70.00	73.68	71.79
**DM**			
control	65.12	70.00	67.47
withdrawal	59.52	62.50	60.98
relapse	53.49	47.92	50.55
**LAT**			
control	48.65	47.37	48.00
withdrawal	54.05	46.51	50.00
relapse	66.67	82.76	73.85
**VM**			
control	50.00	57.14	53.33
withdrawal	50.00	57.14	53.33
relapse	64.71	52.83	57.89
**PC**			
control	44.12	41.67	42.86
withdrawal	54.55	66.67	60.00
relapse	57.58	51.35	54.29

table S2. Morphological alterations of NAc astrocytes predict withdrawal and relapse. Classification performance of Random Forest classifier using the metrics Sensitivity (S), Precision (P) and F1 score for each NAc subdivision, corresponding to heroin (left) or sucrose (right) administration experiments. The classification groups are control, withdrawal and relapse.

a

	Male	Female
**AC**	82.14 ± 2.81	60.74 ± 3.53
**PC**	76.27 ± 2.77	82.21 ± 2.20
**DM**	72.00 ± 1.81	66.70 ± 1.47
**VM**	71.73 ± 3.52	60.54 ± 2.41
**LAT**	63.65 ± 2.11	66.39 ± 3.08

b

	Male				Female			
	Control	Withdrawal	Relapse	Total	Control	Withdrawal	Relapse	Total
**AC**	264	107	96	**467**	44	89	58	**191**
**PC**	551	185	277	**1013**	103	315	122	**540**
**DM**	598	227	345	**1170**	432	681	285	**1398**
**VM**	186	91	124	**401**	81	123	78	**282**
**LAT**	258	91	211	**560**	50	130	132	**312**

table S3. Prediction accuracy in male and female rats. (**a**) High prediction accuracy was observed in both male and female rats across NAc subdivisions. Notable differences in prediction accuracy were observed between males and females in the AC. Number of cells per sex for each brain region and treatment group analyzed is reported in (**b**).

a **Heroin**

	AC					DM					LAT					VM					PC				
	F value	p value	c-w	c-r	w-r	F value	p value	c-w	c-r	w-r	F value	p value	c-w	c-r	w-r	F value	p value	c-w	c-r	w-r	F value	p value	c-w	c-r	w-r
area	5.432	4.575e-03	0.0072	0.992	0.021	25.538	1.042e-11	0.108	0	0	5.572	3.940e-03	0.012	0.012	0.949	3.121	4.473e-02	0.048	0.948	0.135	2.981	5.102e-02	0.205	0.057	0.786
area filled	2.182	1.137e-01	0.1029	0.488	0.773	42.638	6.080e-19	0.002	0	0	3.654	2.629e-02	0.218	0.022	0.754	3.623	2.722e-02	0.020	0.420	0.384	3.474	3.125e-02	0.083	0.847	0.043
equivalent diameter area	3.734	2.441e-02	0.0198	0.343	0.575	59.987	3.459e-26	0.018	0	0	5.859	2.969e-03	0.029	0.004	0.947	4.751	8.936e-03	0.007	0.159	0.520	3.866	2.114e-02	0.055	0.877	0.033
feret diameter max	5.491	4.316e-03	0.0075	0.979	0.017	26.060	6.250e-12	0.168	0	0	9.299	1.009e-04	0	0.003	0.506	4.283	1.418e-02	0.019	0.980	0.050	0.389	6.777e-01	0.891	0.870	0.652
eccentricity	0.908	4.040e-01	0.3879	0.732	0.906	3.612	2.713e-02	0.981	0.033	0.058	0.361	6.968e-01	0.911	0.888	0.677	0.543	5.813e-01	0.983	0.577	0.712	1.350	2.595e-01	0.812	0.228	0.571
perimeter	3.665	2.614e-02	0.0246	0.952	0.127	50.508	3.058e-22	0.014	0	0	4.688	9.436e-03	0.035	0.017	0.999	6.008	2.591e-03	0.002	0.188	0.259	3.977	1.892e-02	0.262	0.286	0.014
perimeter to surface ratio	20.140	3.246e-09	0.9669	0.000	0	13.306	1.783e-06	0.586	0	0	6.823	1.148e-03	0.971	0.002	0.013	0.665	5.148e-01	0.701	0.919	0.500	10.371	3.355e-05	0.002	0	0.695
sphericity	9.280	1.060e-04	0.0013	0.557	0	60.996	1.320e-26	0.102	0	0	5.731	3.366e-03	0.003	0.093	0.287	8.276	2.811e-04	0	0.075	0.204	6.815	1.131e-03	0.085	0.140	0.001
spherical dispropotion	6.782	1.215e-03	0.0084	0.614	0.002	51.566	1.106e-22	0.039	0	0	4.622	1.008e-02	0.010	0.095	0.509	8.040	3.540e-04	0	0.110	0.161	5.748	3.258e-03	0.278	0.081	0.002
solidity	12.933	3.099e-06	0.0172	0.008	0.000	1.990	1.370e-01	0.554	0.508	0.115	3.194	4.148e-02	0.050	0.960	0.081	5.831	3.083e-03	0.003	0.832	0.032	19.305	5.232e-09	0	0	1
major axis	4.277	1.427e-02	0.0815	0.527	0.014	20.958	9.363e-10	0.336	0	0	7.574	5.483e-04	0.001	0.019	0.361	3.200	4.138e-02	0.120	0.841	0.047	0.498	6.079e-01	0.580	0.882	0.904
minor axis	5.054	6.634e-03	0.0136	0.936	0.019	27.744	1.201e-12	0.245	0	0	2.761	6.381e-02	0.244	0.061	0.906	3.681	2.571e-02	0.019	0.454	0.343	5.254	5.321e-03	0.457	0.004	0.111
elogantion	0.991	3.718e-01	0.3392	0.910	0.697	2.789	6.169e-02	0.994	0.074	0.103	0.601	5.483e-01	0.801	0.875	0.517	1.262	2.837e-01	0.647	0.258	0.785	6.154	2.178e-03	0.290	0.001	0.115
fractal dimension	3.067	4.725e-02	0.0386	0.440	0.606	23.716	6.229e-11	0.863	0	0	0.958	3.840e-01	0.364	0.917	0.561	3.143	4.376e-02	0.082	0.089	1.000	8.089	3.201e-04	0.472	0.007	0
lacunarity	0.092	9.119e-01	0.9219	0.946	0.999	11.047	1.671e-05	0.914	0	0	3.795	2.286e-02	0.066	0.036	0.999	0.279	7.565e-01	0.993	0.812	0.770	1.784	1.683e-01	0.149	0.881	0.456

Sucrose

	AC					DM					LAT					VM					PC				
	F value	p value	c-w	c-r	w-r	F value	p value	c-w	c-r	w-r	F value	p value	c-w	c-r	w-r	F value	p value	c-w	c-r	w-r	F value	p value	c-w	c-r	w-r
area	1.364	2.588e-01	0.523	0.255	0.752	30.345	7.481e-13	0	0	0.422	4.239	1.539e-02	0.013	0.166	0.829	5.414	5.517e-03	0.969	0.008	0.012	3.076	4.775e-02	0.124	0.855	0.059
area filled	0.893	4.116e-01	1	0.447	0.438	26.973	1.341e-11	0	0	0.893	2.276	1.047e-01	0.179	0.148	0.909	6.355	2.327e-03	0.956	0.003	0.006	5.063	6.925e-03	0.019	0.944	0.016
equivalent diameter area	1.324	2.691e-01	1	0.301	0.299	27.879	6.145e-12	0	0	0.811	2.017	1.351e-01	0.166	0.245	0.994	6.419	2.194e-03	0.991	0.004	0.005	5.458	4.736e-03	0.045	0.561	0.005
feret diameter max	2.570	7.982e-02	0.254	0.086	0.661	26.934	1.386e-11	0	0	0.030	4.281	1.477e-02	0.017	0.894	0.132	3.636	2.909e-02	0.545	0.022	0.163	1.259	2.856e-01	0.397	0.962	0.326
eccentricity	0.847	4.307e-01	0.770	0.402	0.736	4.397	1.303e-02	0.922	0.021	0.062	5.661	3.900e-03	0.557	0.003	0.029	1.338	2.661e-01	0.428	0.912	0.306	2.676	7.064e-02	0.498	0.373	0.056
perimeter	2.477	8.730e-02	0.994	0.102	0.114	33.177	6.880e-14	0	0	0.623	3.420	3.413e-02	0.324	0.026	0.304	8.069	4.973e-04	0.784	0.004	0.001	10.657	3.481e-05	0.004	0.247	0
perimeter to surface ratio	4.375	1.419e-02	0.856	0.045	0.012	18.629	2.103e-08	0.517	0	0	13.552	2.441e-06	0.263	0.001	0	7.231	1.053e-03	0.002	0.999	0.013	23.432	3.988e-10	0.002	0.000	0
sphericity	4.364	1.434e-02	0.798	0.053	0.012	31.905	2.001e-13	0	0	0.108	5.413	4.947e-03	0.613	0.004	0.030	7.342	9.530e-04	0.296	0.034	0.001	16.131	2.369e-07	0.005	0.012	0
spherical disproportion	4.653	1.091e-02	0.995	0.020	0.014	35.047	1.450e-14	0	0	0.084	5.714	3.705e-03	0.525	0.003	0.030	9.608	1.283e-04	0.256	0.009	0.000	16.519	1.675e-07	0.001	0.037	0
solidity	2.527	8.322e-02	0.382	0.073	0.483	7.453	6.793e-04	0.002	0.003	0.925	7.303	8.126e-04	0.007	0.002	0.595	4.189	1.725e-02	0.735	0.077	0.014	7.215	8.823e-04	0.280	0.036	0.001
major axis	1.051	3.521e-01	0.427	0.457	0.980	21.536	1.563e-09	0	0	0.014	5.205	6.048e-03	0.023	0.919	0.020	2.154	1.202e-01	0.337	0.120	0.699	1.345	2.623e-01	0.345	0.982	0.323
minor axis	2.698	7.052e-02	0.480	0.057	0.343	16.807	1.095e-07	0.000	0	0.590	9.063	1.544e-04	0.005	0.000	0.320	5.254	6.395e-03	0.955	0.008	0.015	7.203	8.927e-04	0.074	0.154	0.001
elogantion	0.814	4.451e-01	0.977	0.544	0.432	4.394	1.306e-02	0.961	0.053	0.024	7.482	6.861e-04	0.645	0.001	0.005	2.116	1.246e-01	0.591	0.455	0.104	3.283	3.898e-02	0.618	0.187	0.031
fractal dimension	1.475	2.320e-01	0.630	0.624	0.211	5.862	3.141e-03	0.022	0.004	0.926	13.439	2.706e-06	0.973	0	0	4.623	1.149e-02	0.012	0.879	0.118	6.178	2.373e-03	0.614	0.023	0.002
lacunarity	6.262	2.428e-03	0.247	0.078	0.002	1.591	2.051e-01	0.696	0.180	0.641	5.539	4.382e-03	0.907	0.019	0.004	5.289	6.190e-03	0.972	0.009	0.013	9.314	1.218e-04	0.005	0.389	0

b **Heroin**

	AC			DM			LAT			VM			PC		
	c-w	c-r	w-r	c-w	c-r	w-r	c-w	c-r	w-r	c-w	c-r	w-r	c-w	c-r	w-r
area	**		*		***	***	*	*		*					
area filled				**	***	***		*		*					*
equivalent diameter area	*			*	***	***	*	**		**					*
feret diameter max	**		*		***	***	***	**		*		*			
eccentricity				*											
perimeter	*			*	***	***	*	*		**					*
perimeter to surface ratio		***	***		***	***		**	*				**	***	
sphericity	**		***		***	***	**			***					***
spherical disproportion	**		**	*	***	***	**			***					**
solidity	*	**	***							**		*	***	***	
major axis			*		***	***	***	*				*			
minor axis	*		*		***	***				*				**	
elongation														**	
fractal dimension	*				***	***								**	***
lacunarity					***	***		*							

Sucrose

	AC			DM			LAT			VM			PC		
	c-w	c-r	w-r	c-w	c-r	w-r	c-w	c-r	w-r	c-w	c-r	w-r	c-w	c-r	w-r
area				***	***		*				**	*			
area filled				***	***						**	**	*		*
equivalent diameter area				***	***						**	**	*		**
feret diameter max				***	***	*	*				*				
eccentricity					*			**	*						
perimeter				***	***			*			**	***	**		***
perimeter to surface ratio		*	*		***	***		***	***	**		*	**	***	***
sphericity			*	***	***			**	*		*	***	**	*	***
spherical disproportion		*	*	***	***			**	*		**	***	**	*	***
solidity				**	**		**	**				*		*	***
major axis				***	***	*	*		*						
minor axis				***	***		**	***			**	*			***
elongation						*		***	**						*
fractal dimension				*	**			***	***	*				*	**
lacunarity			**					*	**		**	*	**		***

table S4. Morphometric alterations during withdrawal and relapse were statistically significant. (**a**) F- and p-values of one-way ANOVA and p-values of the corresponding pairwise tests using Tukey's HSD method to compare the means of control (c), withdrawal (w) and relapse (r) in the five NAc demarcations for each feature during heroin and sucrose administration experiments. The null hypothesis in (**a**) states that means were equal among control, withdrawal and/or relapse in one of the five NAc demarcations for an individual feature. Degrees of freedom between groups was 2 and degrees of freedom within groups was: 656 (heroin AC), 2565 (heroin DM), 869 (heroin LAT), 680 (heroin VM), 1550 (heroin PC), 154 (sucrose AC), 340 (sucrose DM), 275 (sucrose LAT), 130 (sucrose VM), 276 (sucrose PC). (**b**) p-values using Tukey's HSD pairwise tests in (**a**). *p<0.05, **p<0.01, ***p<0.001.

	Heroin				Sucrose			
	Control (µm)	Withdrawal (µm)	Relapse (µm)	Total (µm)	Control (µm)	Withdrawal (µm)	Relapse (µm)	Total (µm)
**AC**	53.723	49.946	53.997	52.657	48.156	44.889	42.703	45.704
**PC**	49.892	50.273	49.435	49.900	45.102	47.014	44.683	45.627
**DM**	47.333	48.148	44.538	46.937	46.084	38.400	40.921	41.682
**LAT**	52.177	47.707	48.925	49.763	47.806	43.975	47.052	45.955
**VM**	55.368	52.093	55.132	54.272	50.098	47.752	42.962	47.585

table S5. Average astrocyte diameter. Diameter measurements reflect the average length of the major axis of the GFAP cytoskeleton.
